# A qualitative analysis of fear of cancer recurrence among Chinese nasopharyngeal cancer patients who are undergoing active treatment

**DOI:** 10.1007/s00520-025-10163-y

**Published:** 2025-11-26

**Authors:** Yanqiu Hu, Maoting Tian, Zhaohui Geng, Katherine Ka Wai Lam, Ting Mao, Qi Liu, Hammoda Abu-Odah, Janelle Yorke, Ka Yan Ho

**Affiliations:** 1 Department of Nursing, Eye & ENT Hospital, Fudan University, Shanghai, China; 2 School of Nursing, Fudan University, Shanghai, China; 3 JBI Fudan University Centre for Evidence Based Nursing, School of Nursing, Fudan University, Shanghai, China; 4 School of Nursing, Shanghai University of Traditional Chinese Medicine, Shanghai, China; 5 School of Nursing, Hong Kong Polytechnic University, Hung Hom, Kowloon, HKSAR China; 6 JC STEM Lab of Digital Oncology Care Enhancement (DOCE), The Hong Kong Jockey Club Charities Trust, Hong Kong SAR, China

**Keywords:** Nasopharyngeal cancer, Fear of recurrence, Qualitative study, Phenomenology

## Abstract

**Purpose:**

Nasopharyngeal cancer (NPC) is one of the most prevalent head and neck cancers. Fear of cancer recurrence (FCR) is a psychological state experienced by cancer patients, characterized by concern and worry about the possibility of cancer recurrence. Long-term and persistently high-level FCR is associated with emotional distress, sleep disorders, and decreased treatment compliance in patients, seriously affecting their quality of life and increasing medical costs. This study employed a phenomenological approach to explore the perceptions of fear of cancer recurrence (FCR) and its impacts on Chinese patients with nasopharyngeal cancer (NPC) who have received radiotherapy.

**Methods:**

We conducted semi-structured interviews, guided by the theoretical model of FCR, with 18 patients diagnosed with NPC at a tertiary hospital in Shanghai, China, between August 2023 and January 2024. All data were transcribed verbatim and then analyzed by two researchers using Colaizzi’s descriptive phenomenological method.

**Results:**

Five themes were identified, including (1) internal triggers of FCR, (2) external triggers of FCR, (3) illness perceptions, (4) challenges and changes due to FCR, and (5) adaptations and responses to FCR.

**Conclusion:**

FCR in patients with NPC is a complex, multidimensional phenomenon involving physiological, psychological, and social dimensions. Our study provides a unique and in-depth understanding of the triggers, challenges, and changes associated with FCR, as well as the adaptations and responses of patients with NPC. We suggest that future research focus on developing appropriate measures and interventions to address FCR in this patient population.

## Background

Nasopharyngeal cancer (NPC) is a malignant tumor that mainly occurs in the top and lateral walls of the nasopharynx and is one of the most common head and neck cancers [1]. Currently, the main treatment for NPC is a combination of radiotherapy, supplemented by chemotherapy [2]. During radiotherapy and chemotherapy, patients may experience multiple symptoms such as hearing loss, tinnitus, visual impairment, and other treatment-related toxicities, as well as emotional distress and various psychological symptoms [3, 4].

Most cancer patients are worried about and afraid of disease progression and recurrence due to the long treatment duration, numerous complications, and high rates of mortality and recurrence associated with cancer [5]. Fear of cancer recurrence (FCR) is a psychological state experienced by cancer patients, characterized by concern and worry about the possibility of cancer recurrence [6]. This psychological state is triggered and maintained by ongoing examinations, increased vigilance, concerns about physical condition, and misinterpretation of normal physical symptoms, such as pain and chest tightness, as signs of cancer recurrence [7]. FCR is a prevalent psychological phenomenon among cancer patients, with 39 to 97% reporting fear of cancer recurrence or tumor progression, and approximately 49% suffering from moderate to severe FCR [6, 8]. FCR can arise at the time of initial cancer diagnosis and persist throughout active treatment and into survivorship trajectories. FCR is reported to be the most common negative emotion among cancer patients and survivors [9], contributing significantly to disease burden and unmet psychological needs [10].

FCR can be explained by the Common-Sense Model of Self-Regulation (CSM) [11]. In this model, an individual’s subjective perceptions of cancer are shaped by their knowledge, illness experience, and treatment history. These perceptions can affect their cognitive and behavioral responses to a cancer diagnosis. When these responses are activated, they lead to corresponding coping strategies and emotional reactions. This process can result in varying levels of FCR, which, in turn, may lead to dysfunctional behaviors such as anxiety, overattention, and over-scrutiny. These behaviors can intensify the fear response and further exacerbate FCR.

Evidence shows that many patients with NPC exhibit a moderate to high level of FCR [12, 13]. Previous studies have also found that patients with NPC experience high levels of FCR during active treatment, which often persist until somatic symptoms diminish several months after treatment completion [12]. Prolonged fear can lead to severe emotional distress, sleep disturbances, and reduced treatment adherence, all of which significantly impair patients’ quality of life and social functioning [14, 15].

According to the Global Cancer Statistics 2020, there were about 133,000 new cases of NPC worldwide in 2020, with China accounting for about 50% of both new cases and deaths [16]. Compared to other common cancers, NPC is anatomically hidden at the back of the nose, making it difficult for patients to detect recurrence. Evidence shows that about 14% of NPC patients experience local or regional recurrence after radical radiotherapy [17]. Recurrent nasopharyngeal carcinoma has a poor prognosis, with a median overall survival of about 20 months [18]. Consequently, patients with NPC often experience higher levels of FCR compared to patients with other common cancers (e.g., bladder, gastric, or lung) [19].

As such, FCR in patients with NPC warrants further exploration to provide a more in-depth understanding to support the development and testing of tailored psychological interventions. The current evidence base consists of two studies that examined FCR and its associated factors among NPC patients; however, these studies employed quantitative methods [12, 20]. To the best of our knowledge, no in-depth qualitative studies have been conducted in this area. To address this gap, this study aimed to explore the experience of FCR in patients with NPC, using an in-depth qualitative approach.

## Methods

### Design

This qualitative study employed a descriptive phenomenological design. Phenomenology is an approach that aims to elucidate the meaning and essence of individuals’ experiences, thereby facilitating understanding [21]. Phenomenology focuses on understanding the world as experienced by the individual, and the researcher seeks to gain a rich and insightful understanding of the phenomenon by setting aside the researcher’s own past experiences and describing the meaning of this experience [21]. The phenomenon of this study was the experience of FCR among NPC patients treated with radiotherapy and/or chemotherapy. This study was conducted following the reporting guidelines outlined in the Standards for Reporting Qualitative Research (SRQR) [22].

### Participants

The study was approved by the Ethics Committee of the Eye and ENT Hospital of Fudan University (2023048) and conducted in accordance with the ethical standards outlined in the Declaration of Helsinki. A purposive sampling method was adopted to recruit NPC patients as participants from a tertiary hospital in Shanghai between August 2023 and January 2024. To achieve maximum variation, participants were purposively chosen based on their age, gender, educational level, work status, tumor stage, and treatment plan. To be eligible, the participants must be (1) at least 18 years old; (2) diagnosed with NPC using pathological assessment; (3) undergoing radiotherapy or chemotherapy, as previous literature indicates that NPC patients receiving active treatment experience high levels of FCR; and (4) able to provide informed consent. Patients were excluded if they had (1) a sudden deterioration of cancer and could not attend the interviews; (2) speech impairments; or (3) were undergoing psychological treatment/intervention. The sample size was based on the principle of data saturation, at which point no new information was extracted from more than three consecutive participants [23]. This indicated that the range of codes or themes had been largely determined, thereby indicating that the study had reached code or theme saturation.

### Data collection

Following informed written consent, two researchers (Hu Yanqiu and Tian Maoting) conducted face-to-face, semi-structured interviews with the participants. They were involved in the daily care of NPC patients and had established a trusting relationship before the interviews began. Several measures were implemented to ensure that the power dynamics would not affect the free expression of patients. First, participants were explicitly informed that their participation was voluntary, and that neither non-participation nor participation would attract any prejudice or benefits to their care. Second, throughout the interviews, researchers closely observed the participants’ non-verbal cues that might indicate hesitancy. For example, if the patients frowned, the researcher would remind them that they could freely express what they wanted, and it would not affect their care. Third, we emphasized that there were no right or wrong answers to any interview questions. Before the commencement of the interview, the researchers explained the purpose and content of the study to the participants and obtained their informed consent. The participants were also informed that the interview would be audio-recorded, and the data collected would be kept confidential and used solely for research purposes. Interviews were conducted in a separate room to ensure a quiet and comfortable environment without being disturbed by other persons and to protect the privacy of the participants. The interviews were conducted according to the semi-structured interview guide designed based on the theoretical model of FCR [11] and relevant literature [24]. The interview guide addressed six different domains, including (1) thoughts and feelings about the disease and treatment, (2) concerns about cancer progression or recurrence, (3) triggers of fears of cancer progression or recurrence, (4) experiences of fears of cancer progression or recurrence, (5) impacts of fears, and (6) management of fears. The interviews were audio-recorded. While the CSM was considered, preconceptions were minimized in the study design by documenting and setting aside CSM-related assumptions before interviews, asking open-ended questions, and using neutral probes that did not reflect researcher assumptions. Field notes were taken to capture non-verbal information such as the participants’ facial expressions and body movements. The interviews were conducted in Mandarin Chinese and lasted between 30 and 40 min. The interviewers and analysts were all trained and familiar with qualitative methods before the study commenced.

### Data analysis and research rigor

The researchers transcribed the audio recordings verbatim. All non-verbal information was marked on the transcripts, supplemented with the field notes. Data were collated and coded using NVivo 14.0 software. Data were analyzed using Colaizzi’s descriptive phenomenological method [25], comprising seven iterative steps: (1) Familiarization: Two researchers (Hu Yanqiu and Tian Maoting) independently conducted repeated line-by-line readings of all interview transcripts to fully understand all contents provided by participants. (2) Identifying significant statements: Both researchers identified verbatim statements directly about FCR. (3) Formulation of meanings: Meaningful fragments were extracted through open coding while considering the field notes. The researchers met to discuss the coding after the first three interviews were processed. Disagreements were resolved through discussion until a consensus was reached. (4) Clustering themes: Meaningful units were clustered into subthemes and synthesized into major themes through constant comparative analysis. (5) Developing an exhaustive description: Themes were rigorously linked to the phenomena of FCR with detailed contextual explanations. (6) Producing the fundamental structure: A comprehensive description of the structure of FCR was presented. (7) Verification: To ensure consistency, the participants were asked to verify that the descriptions reflected their experiences. Specifically, after the interviews, the researchers presented the main findings of the analysis to the interviewees via video conference to verify whether they agreed with the themes and if the themes accurately reflected their feelings and experiences. The participants confirmed that the findings reflected their experiences. To ensure the confirmability and reliability of the findings, a nursing researcher (Geng Zhaohui) with experience studying abroad in English-speaking countries reviewed the documented interpretations to eliminate any potential bias that might have resulted from preconceived ideas and to ensure the validity of the findings. The requirement of studying abroad in English-speaking countries ensured that the nursing researcher had sufficient language proficiency to understand the data and support the translation of findings into English. Also, this researcher supported the translation process from Chinese to English. Following completion of Colaizzi’s steps, we connected themes to the CSM to help contextualize findings rather than force-fit data. This step facilitated the connection of themes to relevant research and theory, allowing for the examination of thematic interactions [26, 27]. To ensure the transferability of this study, the researchers provided a detailed description of the study design and methodology, as well as examples of raw data.

## Results

Eighteen patients with NPC were included and interviewed. There were 11 males and 7 females; their ages ranged from 18 to 63 years (42.83 ± 12.01). Regarding educational attainment, 2 participants had completed primary school, 4 junior high school, 4 high school, 4 junior college, and 4 held bachelor’s degrees. For employment, 10 were employed, and 8 were either unemployed or retired. As for the clinical staging of NPC, 2 were in Stage II, 10 were in Stage III, and 6 were in Stage IV. The characteristics of the participants are presented in Table 1.

**Table 1:** Patient characteristics (*n* = 18)

Item	Variables	% (*n*)
**Age**		42.83 ± 12.01
**Sex**	Male	61.11% (11)
	Female	38.89% (7)
**Marital status**	Married	88.89% (16)
	Single	11.11% (2)
**Education level**	Primary school	11.11% (2)
	Junior high school	22.22% (4)
	High school	22.22% (4)
	Junior college	22.22% (4)
	Bachelor’s degree	22.22% (4)
**Work status**	Employed	55.56% (10)
	Unemployed	38.89% (7)
	Retired	5.56% (1)
**Tumor stage**	Ⅱ	11.11% (2)
	Ⅲ	55.56% (10)
	IV	33.33% (6)
**Place of residence**	Countryside	33.33% (6)
	Town	66.67% (12)
**Treatment**	②	16.67% (3)
	②③	27.78% (5)
	①③	5.56% (1)
	②③④	27.78% (5)
	①②③	5.56% (1)
	②③④⑥	5.56% (1)
	②③⑤	5.56% (1)
	①②③④⑥	5.56% (1)

Treatment: ① Surgery; ② Chemotherapy; ③ Radiotherapy; ④ Biological targeted therapy; ⑤ Immunotherapy; ⑥ Traditional Chinese therapy.

Through the compilation and in-depth analysis of the interview data, with reference to the model of fear of cancer recurrence, five themes were finally formed: internal triggers of FCR; external triggers of FCR; illness perceptions; challenges and changes due to FCR; adaptations and responses to FCR. Table 2 provides a summary of the key themes and subthemes identified in this research.

**Table 2:** The themes and subthemes of FCR

Themes	Subthemes	Quotes	Demographic Profile
Theme 1 Intrinsic stimuli	Subtheme 1 *Uncertainty of disease prognosis*	*“Sometimes I get this pain that makes me feel really on edge… I can’t help but worry—what if it’s spread to my bones?”*	*P13, female, 27 years old, residing in town, bachelor’s degree, employed, married, tumor stage III, receiving chemotherapy and radiotherapy*
Theme 2 External stimuli	Subtheme 1 *Attitudes and concerns of family or friends*	*“It really bothers me a lot. My parents are getting older. I worry that I won’t be able to be there for them. Honestly, the thought of not being able to pull through, scares me. Every time I see my family caring me so much, I fear that my health is declining, and that I will become a burden to them.”*	*Same as above*
	Subtheme 2 *Overwhelmed information*	*“Some patients look like having a hard time. It’s scary to see them having mouth sores due to radiation, which make them unable to eat.”*	*P18, male, 18 years old, residing in town, high school level education, unemployed, single, tumor stage IV, receiving chemotherapy*
	Subtheme 3 *Routine examinations and follow-up*	*“I always feel worry after MRI. I just wonder if the results will be bad.”*	*P6, female, 33 years old, residing in town, high school level education, employed, married, tumor stage III, receiving chemotherapy, radiotherapy, biological targeted therapy, and traditional Chinese therapy*
Theme 3 Illness perceptions	Subtheme 1 Negative treatment experience *Painful treatment experience*	*“The treatment itself is really tough and has a lot of side effects. On top of that, I’m scared that it might come back. What if it does? It’s hard enough now. I don’t know how I can cope if it happens again.”*	*P9, male, 40 years old, residing in town, junior high-level education, unemployed, married, tumor stage III, receiving chemotherapy, radiotherapy, and biological targeted therapy*
	*Fear of adverse reactions from treatment*	*“After my first chemotherapy round, the drugs really suppressed my bone marrow, and my white blood cells were extremely low. I felt so sick and panicked… like the cancer was winning.”*	*P12, female, 57 years old, residing in town, bachelor’s degree, retired, married, tumor stage III, receiving chemotherapy and radiotherapy*
	Subtheme 2 The perceived characteristics of the disease and beliefs about treatments	*“I heard that the cure rate for this is really high, which is pretty reassuring. That’s why I’m not as worried as before.”*	*P18, male, 18 years old, residing in town, high school level education, unemployed, single, tumor stage IV, receiving chemotherapy*
Theme 4 Challenges and changes due to FCR	Subtheme 1 Physiological symptom clusters	*“The second I think about the cancer maybe coming back, I feel scared. Heart racing, gut twisting… Can’t sleep, wake up drenched in sweat with all these worst-case scenarios playing in my head.”*	*P12, female, 57 years old, residing in town, bachelor’s degree, retired, married, tumor stage III, receiving chemotherapy and radiotherapy*
	Subtheme 2 Emotional dysregulation and anxiety about cancer inheritance	*"I looked it up and found that cancer can sometimes run in families. That really makes me worried. Now, every time my child complains of a sore throat, I will worry that something bad may happen.”*	*P1, Male, 51 years old, residing in town, junior college education, employed, married, tumor stage III, receiving chemotherapy and radiotherapy*
	Subtheme 3 Disruption of their daily function and lifestyle practices	*“It’s tough, these thoughts really affect me. For example, you are now employed. But you may have to suddenly take a six-month leave because of recurrence. The job position may be filled by other people, and gone. That thought always bothers me when I have tried to find a job.”*	*P17, male, 40 years old, residing in town, bachelor’s degree, employed, married, tumor stage II, receiving chemotherapy and radiotherapy*
Theme 5 Adaptations and responses to FCR	Subtheme 1 Adopting multiple resources to cope with fear *Seeking medical resources*	*“I checked out cancer hospitals in Beijing and Guangzhou before and during treatment, and ended up choosing this place in Shanghai. Picked the hospital myself, and handpicked my attending doctor too. He’s very experienced, I trust him 100%.”*	*P13, female, 27 years old, residing in town, bachelor’s degree, employed, married, tumor stage III, receiving chemotherapy, radiotherapy, and biological targeted therapy*
	*Seeking family support*	*“I’ve got my family backing me up; they help to keep me going. They are always saying 'Focus on the good, not the what-ifs’… And yeah, they help out with money too.”*	*P9, male, 40 years old, residing in town, junior high-level education, unemployed, married, tumor stage III, receiving chemotherapy, radiotherapy, and biological targeted therapy*
	*Adjusting the social strategies*	*“I can’t handle people trying to cheer me up. It never helps anyway, so I haven’t told anyone. Just the family knows for that reason.”*	*P5, male, 49 years old, residing in town, junior college education, employed, married, tumor stage IV, receiving surgery, chemotherapy, radiotherapy, biological targeted therapy, and traditional Chinese therapy*
	Subtheme 2 Adjustment of lifestyle *Adjusting diet and lifestyle habits*	*“The doctor said no smoked foods, pickled, or spicy things, so I avoid all that.”*	*P4, male, 34 years old, residing in countryside, junior college education, unemployed, married, tumor stage IV, receiving chemotherapy, radiotherapy, and immunotherapy*
	*Avoiding health-compromising physical environments*	*“If someone lights up near me, I’ll say, ‘The smell messes with me since I'm sick.’ If they don’t back off, I’ll try to keep my distance.”*	*Same as above*
	*Exercise to relax the body and mind*	*“I’m practicing Baduanjin to try and get myself into a better place.”*	*P12, female, 57 years old, residing in town, bachelor’s degree, retired, married, tumor stage III, receiving chemotherapy and radiotherapy*
	*Enrich the spare time*	*“Most days I read, and sometimes copy scriptures, such as the Heart Sutra. Calms me down.”*	*P6, female, 33 years old, residing in town, high school level education, employed, married, tumor stage III, receiving chemotherapy, radiotherapy, biological targeted therapy, and traditional Chinese therapy*
	Subtheme 3 Adopting self-regulation strategies *Avoidance of communication about cancer*	*“I keep my issues to myself. What’s the point in telling people? Why bother? It only makes people who hate you to be happy, and hurts the ones who love you. So what’s the purpose of telling other people?”*	*P12, female, 57 years old, residing in town, bachelor’s degree, retired, married, tumor stage III, receiving chemotherapy and radiotherapy*
	*Cognitive avoidance*	*“Sometimes I just want to forget that I am a patient, and then continue my life.”*	*Same as above*
	*Self-consolation*	*“I guess being younger helps, and my body probably fights better than others… So I keep telling myself: the chances of all that happening, super low.”*	*P13, female, 27 years old, residing in town, bachelor’s degree, employed, married, tumor stage III, receiving chemotherapy, radiotherapy, and biological targeted therapy*

### Theme 1: Internal triggers of FCR

#### Subtheme 1: Uncertainty of disease prognosis

Unlike some types of cancer, e.g., breast cancer and skin cancer, NPC develops in anatomically hidden areas like the back of the nose. As such, in the semi-structured interviews, most participants mentioned that they could not conduct self-examinations, e.g., palpation and inspection, to monitor the progress and could only rely on professional examinations to ascertain whether there was cancer recurrence. Furthermore, the treatment period for NPC is lengthy, and the treatment often produces many side effects and complications, such as nasopharyngeal hemorrhage, secretory otitis media, radiation-induced sinusitis, and skull base osteonecrosis. The complications can elicit many physical symptoms, such as pain, bleeding, fatigue, and weakness. Some participants reported that they easily mistook these manifestations for indicators of cancer recurrence or metastasis, thereby exacerbating concerns regarding therapeutic efficacy and uncertainty about prognosis. The emotional distress experienced by them can lead to FCR. This is further illustrated by a participant in the semi-structured interview:

P1: “What worries me is… first, after the treatment, will the cancer actually be gone? Or could it have already spread? Second, will it come back later on? And third, are there any lasting side effects? I know some people bounce back in months, but everyone is different, you know? It really varies.”

### Theme 2: External triggers of FCR

This theme included the attitudes and concerns of family or friends, overwhelmed information, and routine examinations and follow up.

### Subtheme 1: Attitudes and concerns of family or friends

The concern and greetings of family and friends may sometimes become a psychological burden for the patient. NPC patients may feel guilty and blame themselves for being absent from child-rearing or parental support due to the traditional Chinese concept of family responsibility. Some participants mentioned that the care from their families or friends constantly reminded them of their disease status, especially when family members and friends asked the participants about their medical condition and whether the cancer was cured. This triggered the participants to think about the deterioration of the disease and hence increased their vulnerability to FCR.

As illustrated by a participant in the semi-structured interview:

P4: “Sometimes out of the blue, relatives or friends would call and ask how I’m feeling. ‘Any symptoms?’ ‘Have you completed the check at the hospital?’ But every time I talk about my condition, my mind jumps straight to the idea that my cancer may come back… it terrifies me. My kids are just six years old. What if I die? My whole family would fall apart. My wife even told me that if I’m gone, she couldn’t raise both kids on her own. It’s just too much.”

### Subtheme 2: Overwhelmed information

Overwhelming information on the web was also recognized as one of the triggers of FCR. In particular, the participants mentioned that the internet contained a lot of information related to cancer and its treatment, but the accuracy and truthfulness of the information were doubtful. Hence, the participants found it difficult to identify accurate information to assist them in understanding the disease and to guide their decision-making. During the interviews, some participants also mentioned that the personalized information provided by apps further exacerbated their confusion and anxiety because apps would keep presenting similar information based on an individual’s reading history. In the semi-structured interview, a participant stated:

P14: “These days, your phone’s flooded with short videos. Some people claimed that their cancer came back in two months! Once you watch that stuff, the app just keeps showing more to you. Honestly, it really got to me.”

Furthermore, negative cases (e.g., patients with cancer recurrence and/or serious complications) that the participants learned about from their friends, family members, or other patients, or when they witnessed the deterioration of other patients’ health, could intensify their worries, triggering FCR. As illustrated by a participant:

P12: “Once at the clinic, a woman came in, and she couldn't open her mouth due to muscle problems. This really scared me the second I saw that.”

### Subtheme 3: Routine examinations and follow-up

Routine examinations and follow-up visits for NPC were also mentioned by the participants as a trigger of FCR. The participants said they were significantly tense and anxious when they were uncertain and waiting for the results of the examination. In the semi-structured interview, a participant said:

P13: “Right before my monthly check-up, I always get this dreadful feeling… like maybe I'm feeling off, or something’s not right… just no idea what the scan is going to show this time.”

### Theme 3: Illness perceptions

Another theme was illness perceptions, which can be further divided into two categories: (1) negative treatment experiences, which covered painful treatment experiences and fear of adverse reactions from treatment, and (2) the perceived characteristics of the disease and beliefs about treatment.

### Subtheme 1: Negative treatment experience

#### Painful treatment experience

During cancer treatment, the participants experienced a lot of physical and psychological symptoms. The painful treatment experience led the participants to have a deep concern about tumor recurrence and serious side effects from treatment. A participant said in the semi-structured interview:

P14: “After three days of chemotherapy, I felt really bad mentally… just felt like giving up on myself. If the cancer came back, I don't think I could go through it all again.”

### Fear of adverse reactions from treatment

Adverse reactions, e.g., myelosuppression, nausea, and vomiting caused by chemotherapy drugs, not only challenged the participants’ physical health but also increased their level of fear of the treatment and cancer recurrence. This is further supported by a participant in the semi-structured interview:

P12: “After my first chemotherapy round, the drugs really suppressed my bone marrow, and my white blood cells were extremely low. I felt so sick and panicked… like the cancer was winning.”

### Subtheme 2: The perceived characteristics of the disease and beliefs about treatments

Some participants reported that after a certain period, they gained an understanding of the characteristics of their disease and the available treatment options. Subsequently, their confidence in overcoming the disease was also enhanced, thus reducing the fear of recurrence. As mentioned by a participant:

P1: “I went digging online about this disease, and checked out what causes it, treatment options, chemo drugs, different meds, and their side effects. Even looked up the cure rate. Turns out it’s around 90%. Basically, just the common cold of cancers. Haven’t been as scared since.”

### Theme 4: Challenges and changes due to FCR

The fourth main theme identified from the qualitative data was challenges and changes due to FCR, which contained three categories: (1) physiological symptom clusters, (2) emotional dysregulation and anxiety about cancer inheritance, and (3) disruption of daily functioning and lifestyle practices.

### Subtheme 1: Physiological symptom clusters

Some participants said the FCR triggered a range of symptoms, mainly including chest tightness, palpitations, insomnia, night sweats, and lack of concentration. As illustrated by a participant:

P12: “The second I think about the cancer maybe coming back, I feel scared. Heart racing, gut twisting… Can’t sleep, wake up drenched in sweat with all these worst-case scenarios playing in my head.”

### Subtheme 2: Emotional dysregulation and anxiety about cancer inheritance

Some participants said that FCR led to a range of emotional distress, including loss of emotional control, irritability, impatience, or even inexplicable anger towards family members. This is illustrated by a participant in the semi-structured interview:

P11: “The whole tumor-coming-back thing puts me in a bad mood. I’ll blow up at loved ones out of nowhere. Hate that about myself.”

In addition, since some cancers could be genetically inherited, some participants mentioned that their next generation might have cancer. As illustrated by a participant:

P12: “I was worried that my condition might take a bad turn, or worse, become contagious. What if it passes to my kids? One flash of that thought, full-on panic sets in.”

### Subtheme 3: Disruption of their daily function and lifestyle practices

Due to FCR, some participants reported that their work and lifestyle practices were affected. Some participants said they avoided and limited their hobbies to a certain extent as they thought these activities might affect their condition and even lead to cancer recurrence. During the semi-structured interview, one participant said:

P14: “I used to love getting outdoors on holidays. Now, too dare to go. What if someone’s got a cold or something and passes it to me? It could mess with my recovery… And biking, my daughter and I were all about that. But now I’m worried that it might not be good for me, so we’ve stopped.”

### Theme 5: Adaptations and responses to FCR

The last main theme extracted from the data was adaptations and responses to FCR, which contained three subthemes: (1) adopting multiple resources to cope with fear, which covered (i) seeking medical resources, (ii) seeking family support, and (iii) adjusting social strategies; (2) adjustment of lifestyle, including (i) adjusting diet and lifestyle habits, (ii) avoiding health-compromising physical environments, (iii) exercise to relax the body and mind, and (iv) enriching spare time; (3) adopting self-regulation strategies, including (i) avoidance of communication about cancer, (ii) cognitive avoidance, and (iii) self-consolation.

### Subtheme 1: Adopting multiple resources to cope with fear

#### Seeking medical resources

Some participants who were highly educated reported that they actively acquired and learned relevant medical resources, consulted multiple medical institutions for different treatment options, and selected medical teams with extensive clinical experience. Some participants also considered complementary treatments such as traditional Chinese medicine to improve their condition and promote physical recovery through diversified treatment methods. During the semi-structured interview, one participant further illustrated this as follows:

P13: “I checked out cancer hospitals in Beijing and Guangzhou before and during treatment, and ended up choosing this place in Shanghai. Picked the hospital myself, and handpicked my attending doctor too. He’s very experienced, I trust him 100%.”

Besides, some participants with junior college education pointed out that some online resources, e.g., websites of medical institutions and the official WeChat accounts of hospitals, often had professional medical knowledge, making it easier for them to obtain scientific and accurate information about NPC online. As illustrated by a participant:

P5: “I followed the hospital’s WeChat account to get their updates. You know, the scientific stuff. Like if there’s any new findings about my disease, or anything fresh, I’ll see it there.”

### Seeking family support

Some participants mentioned that family support enabled them to effectively manage their FCR. They said a family environment that was characterized by happiness and harmony could have a positive impact on them. In particular, the happy and harmonious family environment could enhance their belief in the efficacy of treatment. During the semi-structured interview, a participant said:

P9: “I’ve got my family backing me up; they help to keep me going. They are always saying ‘Focus on the good, not the what-ifs’….And yeah, they help out with money too.”

Moreover, the experiences of family members or friends who have successfully fought against cancer could serve as a source of inspiration for patients, providing them with a sense of spiritual support. In addition, the companionship of pets could provide comfort and relieve stress for participants. As one of the participants said:

P13: “When I break down crying, my family consoles me, and puts me back together… And my uncle, he’s an NPC survivor going strong for over ten years now. Gives me real faith in beating this.”

### Adjusting the social strategies

Some participants said they had to strategically adjust their social activities by actively participating in various leisure activities, such as watching others play chess, chatting, and dining with colleagues or friends to relieve their bitterness. As supported by one participant in the semi-structured interview:

P4: “When I’m alone, my mind races. Got to distract myself, ah, like watching folks play chess. Way better… or just chatting with others about anything light.”

In addition, some participants said they might avoid social gatherings, selectively disclose their disease status, and share their conditions only with their closest relatives. All of these could help reduce the psychosocial pressure caused by the disease. As one of the participants said:

P5: “I can’t handle people trying to cheer me up. It never helps anyway, so I haven’t told anyone. Just the family knows for that reason.”

### Subtheme 2: Adjustment of lifestyle

#### Adjusting diet and lifestyle habits

A majority of participants said they changed some bad habits that were believed to be related to cancer recurrence. These habits included eating spicy foods and/or pickled foods, staying up late, smoking, and drinking alcohol, etc. Some participants also said they reduced their consumption of takeout food and chose to cook for themselves to ensure that the food was healthy. As illustrated by a participant:

P4: “The doctor said no smoked foods, pickled, or spicy things, so I avoid all that.”

#### Avoiding health-compromising physical environments

Some participants mentioned that they would avoid exposure to health-compromising environments, such as keeping an appropriate distance from colleagues who smoked to reduce the chance of passive smoking. As illustrated by one participant in the semi-structured interview:

P4: “If someone lights up near me, I’ll say, ‘The smell messes with me since I’m sick’. If they don’t back off, I’ll try to keep my distance.”

#### Exercise to relax the body and mind

Some participants said they had participated in some aerobic exercise, such as walking, cycling, and practicing Baduanjin, to relax their bodies and minds. During the semi-structured interview, one participant said:

P9: “I would take a walk, and it would settle things down gradually.”

#### Enrich the spare time

Some participants also mentioned that they had engaged in various hobbies, such as reading, copying Buddhist scriptures, watching films, and traveling,. This could enrich their spare time and assist them in effectively relieving psychological pressure. During the interview, a participant said:

P6: “Most days I read, and sometimes copy scriptures, such as the Heart Sutra. Calms me down.”

### Subtheme 3: Adopting self-regulation strategies

#### Avoidance of communication about cancer

Most participants said they usually concealed their FCR deep within themselves and coped with these negative emotions independently rather than seeking support from others.

Besides, some participants said they believed that cancer was “destiny” or a fate that could not be changed. Some also mentioned that it was unnecessary to seek help from others, as other people could not understand their feelings or provide substantial assistance. This is illustrated by a participant in the semi-structured interview:

P1: “What’s meant to be will be. No use overthinking it, and no need to talk it out.”

#### Cognitive avoidance

Some participants said they attempted to avoid negative emotions by consciously avoiding negative information on the internet and trying to forget their condition. Some participants also said they distracted themselves by watching short videos or committing themselves to work to avoid repeated or recurring negative thoughts. One participant said:

P6: “See anything negative, my brain auto-blocks it. Only let the good stuff in.”

#### Self-consolation

Some participants told us that they had been confronted with worries related to cancer recurrence by presenting a seemingly positive outlook through denying unfavorable information and constantly reassuring themselves. They would comfort themselves that the probability of tumor recurrence was very low and list various factors that could reduce the risk of recurrence, such as young age and a good immune system. As illustrated by one participant in the semi-structured interview:

P13: “I guess being younger helps, and my body probably fights better than others… So I keep telling myself: the chances of all that happening, super low.”

## Discussion

Fear of cancer recurrence is a common phenomenon among cancer patients, including those diagnosed with NPC. Existing literature on FCR in NPC patients primarily examines the prevalence and its associated factors but has neglected the voices of patients, especially their experiences of FCR when receiving active treatment. To bridge the gap in the existing literature, we conducted a qualitative study to explore and summarize the experiences of FCR in Chinese patients with NPC. In addition, applying the CSM, a widely used theoretical framework that explains how individuals perceive and respond to health threats, we identified the triggering factors, patients’ perceptions and emotional reactions, challenges and changes, and patients’ coping strategies for FCR based on unique qualitative responses.

As our study revealed, diagnostic procedures and disease-related factors, such as the occurrence of various symptoms during active treatment and uncertainty about the disease prognosis, were significant triggers of FCR in NPC patients. In particular, some participants reported that treatment-related toxicities, such as nasopharyngeal hemorrhage, secretory otitis media, radiation-induced sinusitis, and skull base osteonecrosis, elicited physical symptoms such as pain, bleeding, fatigue, and weakness. They easily mistook these manifestations for indicators of cancer recurrence or metastasis, increasing anxiety and worry, eventually leading to FCR. Our results are consistent with the quantitative findings of Tsay et al. [28], which showed that treatment-related somatic symptoms, such as pain in the mouth, throat, and neck, were associated with higher levels of FCR in patients with head and neck cancer. When compared to other cancer types, patients with NPC often experience multiple symptom clusters, including fatigue-sleep-emotion, oral mucosal, vocal-dysphagia, and gastrointestinal symptom clusters during chemoradiotherapy [4, 29]. With the accumulation of radiation doses and chemotherapeutic agents, the number and severity of symptoms significantly increase when the treatment continues [29]. Therefore, interventions to manage FCR in patients with NPC should be initiated during the mid-phase of treatment.

Our semi-structured interview found that the uncertainty of the disease could also trigger FCR in NPC patients. The uncertainty mainly came from the inability to predict the prognosis of the disease, the lack of information related to the diagnosis and severity of the disease, and vague symptoms. Dodd, in the theory of symptom management, emphasized [30] that the priority in symptom management is to gain a comprehensive understanding of the patients’ experience of symptoms, and this entails paying close attention to the patients’ perception of their symptoms, assessments, and reactions. Therefore, when developing appropriate interventions for FCR, it is imperative to comprehend the triggers of FCR, support patients in managing symptom clusters, enhance their understanding of physical symptoms, and reduce disease uncertainty, thus reducing the triggers of FCR. There is increasing evidence that electronic platforms, such as the internet, may assist patients in managing symptoms and alleviating their suffering [31]. Specifically, some electronic platforms have been developed in the West to educate patients with various physical symptoms and offer personalized information to help alleviate their fears or worries caused by somatic symptoms [32, 33]. This could be a potential option for healthcare professionals to consider as an intervention to manage and reduce FCR in patients with NPC.

Access to accurate information about the disease and its symptoms is important for alleviating FCR among patients with NPC. Our qualitative interview showed that new media in the Chinese context, e.g., WeChat, Baidu, Xiaohongshu, and TikTok, were an important way for our participants to access support, especially those with higher educational backgrounds or living in urban areas, to obtain the information. This is likely because the study hospital was in an urban area where well-developed access to online educational resources was available for patients. Hence, NPC patients in urban areas are likely to receive disease-related information through new media platforms. Although information provision via different sources is important, excessive information can also overwhelm patients and trigger FCR, especially when the patients are unable to distinguish the reliability of such information [34].

This is further illustrated by our participants in the semi-structured interviews who shared that negative and/or misleading information exacerbated their fear about the deterioration of their medical condition. This is now even more common due to the development of natural language processing, which is a computational technique used to identify the users’ search preferences and lead to the appearance of more similar information according to the search history [35]. Hence, cancer patients who read or search for some negative information, e.g., cancer metastasis online, will be continuously bombarded with similar negative information, potentially increasing their psychological burden and FCR [36]. To address this issue, it is vital to provide patients with correct information related to cancer and its treatment. Specifically, healthcare professionals should optimize patients' access to and management of online information and assist them in developing a comprehensive understanding of their disease, thereby reducing the impact of misleading information. This can reduce patients’ concerns about their conditions and encourage them to engage more actively in the self-management of their disease.

In this study, some NPC patients were able to take the initiative to obtain medical resources, seek family and social support, and adjust their lifestyles and social activities to cope with the challenges associated with FCR. However, we observed that most participants relied on negative coping strategies, such as cognitive avoidance and self-comfort, to manage FCR. This may, in part, be due to the influence of Confucianism, which emphasizes forbearance, a concept that everyone should take control of their emotions [37]. As such, patients with NPC may feel reluctant to express their feelings and emotions to others. Self-disclosure refers to the process of sharing personal experiences, feelings, and thoughts with others, which helps individuals release suppressed emotions, change their understanding of stressful events, enhance intimate relationships with others, and gain social support, thereby promoting individuals’ adjustment and adaptation to stressors [38]. A previous study indicated a negative correlation between self-disclosure and the level of fear of recurrence in patients with nasopharyngeal carcinoma [39]. Given the potential therapeutic value of self-disclosure, encouraging expression may enhance emotional self-regulation abilities and alleviate FCR. Another study that examined the use of self-disclosure in couples with breast cancer showed that this intervention could improve the negative emotions of breast cancer patients and effectively reduce their FCR [40]. Nevertheless, recent studies on self-disclosure have mainly been conducted in breast cancer patients, and the intervention components have mostly focused on written expression and face–to–face interviews with husbands and wives [40, 41]. In the future, healthcare professionals and researchers can consider modifying the existing interventions on self-disclosure according to the specific coping characteristics of NPC patients to address their FCR.

Our findings align with and extend the CSM. While we observed expected correspondences between participants’ experiences and CSM domains, with triggers of FCR (intrinsic/external stimuli) mapping to illness stimuli, illness perceptions reflecting cognitive/emotional representations, and coping responses aligning with emotion-focused strategies, our findings contribute a critical advancement. The theme “Challenges and changes due to FCR” demonstrated that the impact of recurrence fear extends far beyond immediate health concerns, manifesting in three underrecognized dimensions: (1) physiological symptom clusters (e.g., insomnia, palpitations), which highlight the somatic embedding of sustained distress; (2) negative psychological symptoms (e.g., emotional dysregulation, transgenerational anxiety), revealing how FCR disrupts core aspects of mental well-being; (3) limitations on personal interests and development (e.g., abandoned careers, restricted hobbies), illustrating profound biographical disruptions that can persist long after treatment completion. These findings extend the CSM’s framing of “consequences” by showing how FCR reshapes survivors’ lives across physiological, psychological, and existential dimensions.

### Limitations

The participants in this study were recruited from a single hospital, which may limit the representativeness. Future studies may consider recruiting participants from multiple centers across diverse geographic regions to enhance the representativeness of the sample. Additionally, this study employed a cross-sectional qualitative design. Longitudinal studies are recommended to explore how FCR experiences evolve from treatment into survivorship.

## Conclusion

This study revealed that FCR in patients with NPC was triggered by various intrinsic and extrinsic factors. The impact of FCR was not limited to the physical symptoms experienced by patients, but also led to complex psychological distress, social isolation, and impairments in personal development. Most patients tended to regulate their emotions independently when facing FCR and were unwilling to express or share negative emotions with others. To address FCR, healthcare professionals should assist patients in developing a correct understanding of the disease and its treatment, particularly given the proliferation of misleading cancer-related information online. Additionally, future studies should explore the potential therapeutic value of interventions on self-disclosure in patients with NPC to help them manage FCR throughout their treatment trajectory.

## Data Availability

The datasets used and/or analyzed in this study can be requested from the corresponding author for reasonable cause, subject to the approval of the ethics committee that approved the original study.
